# Cryptic biodiversity and phylogeographic patterns of Seychellois *Ligia* isopods

**DOI:** 10.7717/peerj.3894

**Published:** 2017-10-06

**Authors:** Carlos A. Santamaria, Joanna K. Bluemel, Nancy Bunbury, Melinda Curran

**Affiliations:** 1 Biology Faculty, College of Science and Mathematics, University of South Florida Sarasota-Manatee, Sarasota, FL, United States of America; 2 Department of Biological Sciences, Sam Houston State University, Huntsville, TX, United States of America; 3 Marine Conservation Society Seychelles, Mahé, Seychelles; 4 Lowestoft Laboratory, Centre for Environment, Fisheries and Aquaculture Science (CEFAS), Lowestoft, Suffolk, United Kingdom; 5 Seychelles Islands Foundation, Mahé, Seychelles; 6 Island Conservation Society, Mahé, Seychelles

**Keywords:** Western Indian Ocean biogeography, Oniscidea, Cryptic species, Ligiidae, Intertidal, Overwater dispersal, Sea-land interphase, Vicariance

## Abstract

*Ligia* isopods are conspicuous inhabitants of rocky intertidal habitats exhibiting several biological traits that severely limit their dispersal potential. Their presence in patchy habitats and low vagility may lead to long term isolation, allopatric isolation and possible cryptic speciation. Indeed, various species of *Ligia* have been suggested to represent instead cryptic species complexes. Past studies; however, have largely focused in Eastern Pacific and Atlantic species of *Ligia*, leaving in doubt whether cryptic diversity occurs in other highly biodiverse areas. The Seychelles consists of 115 islands of different ages and geological origins spread across the western Indian Ocean. They are well known for their rich biodiversity with recent reports of cryptic species in terrestrial Seychellois organisms. Despite these studies, it is unclear whether coastal invertebrates from the Seychelles harbor any cryptic diversity. In this study, we examined patterns of genetic diversity and isolation within *Ligia* isopods across the Seychelles archipelago by characterizing individuals from locations across both inner and outer islands of the Seychelles using mitochondrial and nuclear markers. We report the presence of highly divergent lineages of independent origin. At Aldabra Atoll, we uncovered a lineage closely related to the *Ligia vitiensis* cryptic species complex. Within the inner islands of Cousine, Silhouette, and Mahé we detected the presence of two moderately divergent and geographically disjunct lineages most closely related to *Ligia dentipes*. Our findings suggest that the Seychelles may harbor at least three novel species of *Ligia* in need of description and that these species may have originated independently.

## Introduction

**Figure 1:**
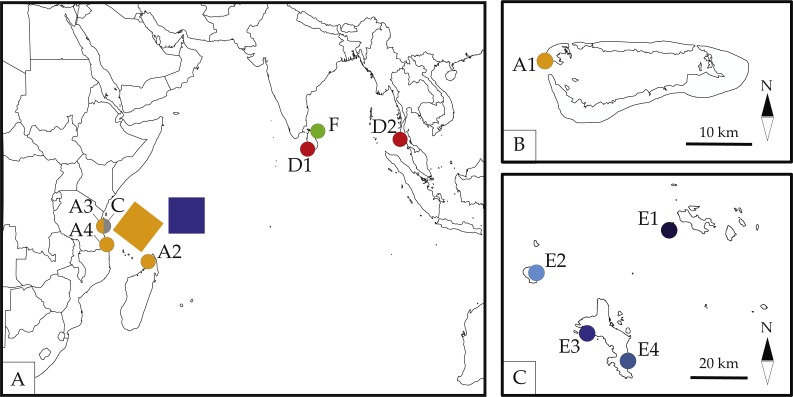
Map of sampled localities across the Seychelles. A1-Basin Cabri, Aldabra Atoll, Seychelles; A2-Nosy Be, Madgascar; A3-The Slipway, Dar-Es-Salaam, Tanzania; A4-Kilwa Masoko, Tanzania; C-Stone Town, Zanzibar, Tanzania; D1-Galle, Sri Lanka; D2-Patong Bay, Phuket, Thailand; E1-Cousine Island, Seychelles; E2-Silhouette Island, Seychelles; E3-L’Islette, W. Mahé, Seychelles; E4-Anse Parnel, S.E. Mahé, Seychelles; F-Dutch Bay, Trincomalee, Sri Lanka. Colors and labels for each locality correspond with other figures and tables. Detailed information for each locality is presented in Table 1. (A) Map by Ding Zhiren under a CC BY-SA 3.0 license; (B) map by Ewan ar Born under a CC BY-SA 3.0 license; (C) Map in the public domain in Wikimedia Commons at https://commons.wikimedia.org/wiki/File:Seychelles_large_map.jpg.

The Seychelles encompass some 115 islands spread across a large swath of the western Indian Ocean. These islands can be broadly divided into outer and inner islands based on their geological origin, geographic locality, and unique geological histories (Braithwaite, 1984). The “outer” islands are low-lying coralline islands that vary in size, age and geographic distribution. Some outer islands, such as Aldabra Atoll and the Farquhar group, lay closer to Africa and Madagascar than to other islands in the Seychelles. The inner islands, on the other hand, consist of granitic islands thought to have formed as the Indian and Madagascar Plates separated during the breakup of Gondwana some 65 million years ago (Mya) (Plummer & Belle, 1995). The inner islands comprise of some 40 islands, including major ones such as Mahé, Praslin and Silhouette as well as smaller islands encircling them (Fig. 1). The complex geological history of the Seychelles islands coupled with their remoteness may help explain a richly biodiverse fauna characterized by high rates of endemism and for the presence of highly divergent cryptic lineages recently reported across a variety of Seychellois terrestrial (e.g., Rocha, Harris & Posada, 2011; Rocha, Posada & Harris, 2013; Silva et al., 2010) and freshwater (e.g., Daniels, 2011) organisms. These recent reports indicate that our understanding of the Seychelles fauna may be incomplete and that cryptic species may exist in Seychellois organisms from poorly studied habitats. This is particularly true for organisms in which cryptic lineages have been reported from other highly isolated archipelagos.

*Ligia* isopods have been reported to exhibit high levels of cryptic diversity in several regions of the world, including highly isolated locations such as the Hawaiian islands (Santamaria et al., 2013; Taiti et al., 2003). In this remote and isolated archipelago, *Ligia hawaiensis* was thought to be the only intertidal species of this genus to occupy this habitat (Schmalfuss, 2003). This species, however, appears to represent a paraphyletic taxon composed of several highly divergent (>10% COI K2P) lineages (Santamaria et al., 2013). These results indicate that this species may actually consist of several cryptic species. The high levels of divergence observed in *L. hawaiensis* match those reported in other *Ligia* species (Eberl et al., 2013; Hurtado, Mateos & Santamaria, 2010; Jung et al., 2008; Santamaria, Mateos & Hurtado, 2014; Santamaria et al., 2013; Taiti et al., 2003) and are thought to be a product of their biological traits that severely restrict dispersal abilities: poor desiccation resistance, poor ability to swim, direct development, and inhabitance of patchy rocky habitats. Such traits, when combined with the isolated nature of remote oceanic archipelagos can easily disrupt gene flow leading to local speciation, and possibly cryptic speciation, in populations. This leads to the question of whether *Ligia* populations in isolated and remote islands such as those of the Seychelles may also harbor cryptic lineages.

In the Seychelles, only two species of *Ligia* isopods have been formally reported to date (Ferrara & Taiti, 1985; Taiti, 2014). The earliest published record from the Seychelles archipelago indicates the presence of *L. exotica*, a cosmopolitan species of Asian origin, at Aldabra (Ferrara & Taiti, 1985). This record is based on a single 8-mm long juvenile specimen collected at Aldabra Atoll in 1983. More recently, the presence of *L. dentipes*, a species widely distributed through the Indian Ocean and redescribed and illustrated by Ferrara & Taiti (1982), from the island of Silhouette was reported (Taiti, 2014). These reports are based on morphological identification and in some cases of partial or incomplete organisms, casting doubt on some of the identifications (e.g., *L. exotica* from Aldabra) and leaving unanswered whether Seychelles *Ligia* harbor highly divergent lineages. In this study, we apply molecular and morphological approaches to *Ligia* individuals collected in both the inner and outer islands to determine which *Ligia* species inhabit the Seychelles and whether they harbor any highly divergent cryptic lineages that may present putative cryptic species.

**Table 1:** Localities included and corresponding GenBank Accession Numbers for all genetic markers used, latitude, and longitude. Map labels correspond with other figures and tables.

Species	Locality	Map label	16S Acc. no.	12S Acc. no.	COI Acc. no.	Cytb Acc. no.	NaK Acc. no.	Latitude	Longitude
*L. vitiensis*	Basin Cabri, Aldabra Atoll, Seychelles	A1	MF828579	MF828590	MF805571 MF805572	MF805561	MF805565	N/A	N/A
*L. vitiensis*	Nosy Be, Madgascar	A2	AY051342	N/A	AY051323	N/A	N/A	N/A	N/A
*L. vitiensis*	The Slipway, Dar-Es-Salaam, Tanzania	A3	MF828580	MF828591	MF805569	MF805562	N/A	6°45′06.9″S	39°16′18.6″E
*L. vitiensis*	Kilwa Masoko, Tanzania	A4	MF828581	MF828592	MF805570	MF805563	N/A	8°55′42.7″S	39°31′17.9″E
*L. vitiensis*	Dili, Timor-Leste	N/A	KF546556	KF546581	KF546662	KF546725	N/A	N/A	N/A
*L. vitiensis*	Labuanbajo, Flores, Indonesia	N/A	KF546555	KF546580	KF546663	KF546724	N/A	N/A	N/A
*L. vitiensis*	Stone Town, Zanzibar, Tanzania	C	MF828578	MF828589	MF805568	MF805560	N/A	6°09′33.8″S	39°11′26.4″E
*L. dentipes*	Galle, Sri Lanka	D1	MF828576	MF828584	MF805566	MF805557	N/A	6°01′49.9″N	80°13′03.3″E
*L. dentipes*	Patong Bay, Phuket, Thailand	D2	KF555801	KF555838	KF555841	KF555754	N/A	7°53′11.0″N	98°17′10.3″E
*L. dentipes*	Cousine Island, Seychelles	E1	MF828572	MF828585	MF805578 MF805579 MF805580 MF805581 MF805582 MF805583 MF805584	MF805559	N/A	4°20′55.3″S	55°38′41.9″E
*L. dentipes*	Silhouette Island, Seychelles	E2	MF828573	MF828586	MF805573 MF805574 MF805575	MF805558	MF805564	4°29′08.4″S	55°15′12.4″E
*L. dentipes*	L’Islette, W. Mahé, Seychelles	E3	MF828574	MF828587	MF805576 MF805577	N/A	N/A	4°39′46.7″S	55°24′35.0″E
*L. dentipes*	Anse Parnel, S.E. Mahé, Seychelles	E4	MF828575	MF828588	MF805585 MF805586	N/A	N/A	4°46′01.1″S	55°31′19.3″E
*L. dentipes*	Dutch Bay, Trincomalee, Sri Lanka	F	MF828577	MF828583	MF805567	MF805556	N/A	8°33′52.6″N	81°14′27.8″E
*L. occidentalis*	Guaymas, Mexico	N/A	KF546553	KF546583	KF546666	KF546728	N/A	27°54′44.3″N	110°56′49.6″W
*L. exotica*	Veracruz, Mexico	N/A	KF546552	KF546584	KF546664	KF546726	N/A	19°11′40.2″N	96°07′24.4″W

## Materials and Methods

We collected *Ligia* individuals by hand in locations in the inner and outer islands of the Seychelles Archipelago, Zanzibar and mainland Tanzania, and Sri Lanka. All specimens were collected during 2014, field-preserved in 70% Ethanol, and frozen upon arrival at the laboratory. Collections in the Seychelles were carried out under Seychelles Bureau of Standards permit A0157. The data produced from these samples was complemented using publicly available sequences for four *Ligia* species: *L. occidentalis* (Mexico), *L. exotica* (Mexico), *L. dentipes* (Thailand), and *L. vitiensis* (Indonesia, Timor-Leste, Madagascar). Detailed information for all localities is provided in Table 1.

We extracted total genomic DNA from pereopods/pleopods for 10 individuals per locality following the solid tissue protocol of the ZR Quick-gDNA Miniprep Kit (Zymo Research, Irvine, CA, USA). During dissections, male specimens were identified to species by visually inspecting the appendix masculina of the second pleopod and comparing it to illustrations from species descriptions and re-illustrations (Ferrara & Taiti, 1982; Khalaji-Pirbalouty & Wägele, 2010; Taiti et al., 2003; Taiti, Ferrara & Kwon, 1992). For *Ligia* collected in the Seychelles, we PCR amplified a 710-bp segment of the Cytochrome Oxidase I mitochondrial gene (hereafter COI) and a 710-bp fragment of the *α*-subunit of the Sodium-Potassium ATPase gene (hereafter NaK) for 1–10 individuals per locality using previously published primers and conditions (COI: Folmer et al., 1994; NaK: Tsang et al., 2008). We PCR-amplified an additional three mitochondrial genes for a subset of individuals from each locality in the Seychelles (see Fig. 1, Table 1): (a) ∼490-bp of the 16S rDNA gene (primers 16Sar/16Sbr; Palumbi, 1996); ∼495-bp of 12S rDNA (primers crust-12Sf/crust-12Sr; Podsiadlowski & Bartolomaeus, 2005); and a 361-bp fragment of the Cytochrome-b (Cytb) gene (primers 144F/151F and 270R/272R; Merritt et al., 1998). For *Ligia* from localities outside the Seychelles, we amplified all four mitochondrial genes mentioned above for 1–2 individuals per locality (see Table 1). Positive PCR amplicons were identified using gel electrophoresis prior to cleaning and sequencing at the University of Arizona Genetics Core (UAGC). Sequences were assembled and edited (i.e., primers removed) using Geneious R8.0.2. No evidence indicative of pseudo-genes (i.e., no gaps, indels, early stop codons) was observed in any of the protein coding genes used in this study (i.e., NaK, cyt-b, and COI).

We used TCS v1.21 (Clement, Posada & Crandall, 2000) to visualize the relationships between COI haplotypes recovered from *Ligia* from all Seychelles localities to both determine the geographic distribution of COI haplotypes recovered within the archipelago and the levels of fine scale divergence within the archipelago. We calculated the 95% most parsimonious branch connections between haplotypes under the cladogram estimation algorithm of Templeton, Crandall & Sing (1992) with all other settings as default. We also calculated Kimura 2-Parameter distances (K2P) within and between localities for COI in MEGA v7.0.7 (Kumar, Stecher & Tamura, 2016). We estimated relationships between NaK alleles using a similar approach.

We combined the four mitochondrial gene fragments produced in this study with publicly available sequences for other *L. vitiensis* from the Indo-Pacific and for two other *Ligia* species to be used as outgroups (i.e., *L. occidentalis* and *L. exotica* collected in Mexico). Locality and sequence information for these sequences is presented in Table 1. Since ribosomal genes used in this study exhibit secondary structure that may lead to ambiguous alignments, we aligned each gene dataset separately using MAFFT v.7.0 (Katoh & Standley, 2013) as implemented under the GUIDANCE2 algorithm (Sela et al., 2015) in the GUIDANCE server (http://mafft.cbrc.jp/alignment/server/) using 100 bootstrap replicates and all other settings as default. This approach produces confidence scores for each nucleotide position and sequence in the alignment that aid in the non-biased removal of misaligned nucleotide positions. We excluded nucleotide positions from our final alignment if they: (a) produced a confidence score below 1.00 in the Guidance alignments, and/or (b) if they exhibited obvious misalignments. We used MEGA v7.0.7 (Kumar, Stecher & Tamura, 2016) to estimate pairwise K2P genetic distances for COI and for the 16S rDNA genes, after excluding ambiguously aligned sites.

We used jModeltest v2.1 (Darriba et al., 2012) to determine the most appropriate model of nucleotide evolution for the final concatenated mitochondrial alignment as well as for each mitochondrial gene. We selected from 1,624 models by evaluating their likelihoods on a fixed BioNJ-JC tree under the Bayesian Information Criterion (BIC). We used the most likely model of nucleotide evolution chosen by jModeltest in our phylogenetic reconstructions with two general exceptions. When the chosen model was not implemented by the software, we applied the next more complex model implemented. When the chosen model implemented the joint estimation of Γ and I, we used a simpler +Γ model as the joint estimation of Γ and I can be problematic (see RAxML manual; and pages 113–114 of Yang, 2006).

We carried out Maximum Likelihood phylogenetic reconstructions in RAxML v8.2.6 (Stamatakis, 2014; Stamatakis, Hoover & Rougemont, 2008) and GARLI v2.0 (Zwickl, 2006). In RAxML, we used the Rapid Bootstrap Algorithm to carry out 1,000 bootstrap replicate searches followed by a thorough ML search under the GTR +Γ model. All other settings were as default. Searches in GARLI consisted of 1,000 bootstrap replicates using the appropriate model of evolution identified by jModeltest, with all other settings as default. For each search, we produced a majority-rule consensus tree of all bootstrap replicates using the SumTrees command of DendroPy v3.10.1 (Sukumaran & Holder, 2010).

**Table 2:** Settings for Maximum Likelihood and Bayesian analyses for the concatenated mitochondrial dataset.

Software	Model & priors^a^	Part scheme^b^	Iterations gen./bootstrap replicates	Sample freq	Runs/ chain	Burnin	ASDSF^c^	Bayes factor/ML scores (−lnL)^d^	ESS > 200^e^	PSRF^f^
RAXML	GTR +Γ	Unpart	1,000	n/a	n/a	n/a	n/a	−9006.5300	n/a	n/a
RAXML	GTR +Γ	Gene	1,000	n/a	n/a	n/a	n/a	−8865.7562	n/a	n/a
RAXML	GTR +Γ	BP	1,000	n/a	n/a	n/a	n/a	−8334.7701	n/a	n/a
Garli	012010 + Γ + *F*	Unpart	1,000	n/a	n/a	n/a	n/a	−8986.6239	n/a	n/a
Garli	Mixed model	Gene	1,000	n/a	n/a	n/a	n/a	−8972.0488	n/a	n/a
Garli	Mixed model	BP	1,000	n/a	n/a	n/a	n/a	−8212.4919	n/a	n/a
MrBayes	GTR +Γ	Unpart	2 × 10^8^	5,000	4	25%	0.000724	−8959.8260	Yes	1
MrBayes	GTR +Γ	Gene	2 × 10^8^	5,000	4	25%	0.001157	−8928.4492	Yes	1
MrBayes	GTR +Γ	BP	2 × 10^8^	5,000	4	25%	0.001170	−8608.5042	Yes	1
Phycas	GTR +Γ	Unpart	1 × 10^6^	50	n/a	25%	n/a	−8959.6281	Yes	n/a
Phycas	GTR +Γ	Gene	1× 10^6^	50	n/a	25%	n/a	−8879.1040	Yes	n/a
Phycas	GTR +Γ	BP	1 × 10^6^	50	n/a	25%	n/a	−8324.1730	Yes	n/a

**Notes.**

a All others default.

b BP as indidcated by PartitionFinder (1, 16S +12S +Cytb 2nd codons; 2, COI 1st codons + Cytb 1st codons; 3, COI 2nd codons; 4, Cytb 3rd codons + COI 3rd codons).

c Average standard deviation of split frequencies.

d Estimated in Tracer v.1.5.

e Effective sample size.

f Potential Scale Reduction Factor for all parameters.

We also carried out phylogenetic reconstructions under Bayesian Inference. Parameters used in MrBayes v3.1.2 (Huelsenbeck & Ronquist, 2001; Ronquist & Huelsenbeck, 2003) searches are presented in Table 2. All other parameters used were as default. We also carried out Bayesian searches implementing polytomy priors (Lewis, Holder & Holsinger, 2005) under Phycas v2.2.0 (Lewis, Holder & Swofford, 2015) as to ensure that support values produced by MrBayes were not overestimated (i.e., “star-tree paradox”) (Suzuki, Glazko & Nei, 2002). We determined if Bayesian analyses had reached stationarity prior to estimating the posterior probability for each node by building majority-rule consensus trees of the stationary stage of each run using the SumTrees command (Sukumaran & Holder, 2010). Samples prior to stationarity were discarded as “burnin”.

We repeated all phylogenetic searches under three partitioning schemes: (a) all positions within a single partition; (b) positions partitioned by gene; and (c) the best partitioning scheme according to the BIC implemented in PartitionFinder v1.0.0 (Lanfear et al., 2012). PartitionFinder searches were run using the following settings: branch lengths = linked; models = all; model selection = BIC; search = greedy; and an *a priori* partitioning scheme accounting for codon positions and genes.

## Results

We sequenced COI and NaK for a total of 50 individuals from across five locations in the Seychelles archipelago. All individuals from the inner islands of the Seychelles were putatively identified as *L. dentipes* based on the gonopod morphology, while those from Aldabra were putatively assigned to *L. vitiensis*. All sequences produced in this study have been deposited in GenBank under accession numbers: MF805556–MF805586 and MF828572–MF828583.

### COI and NaK haplotype networks

**Figure 2:**
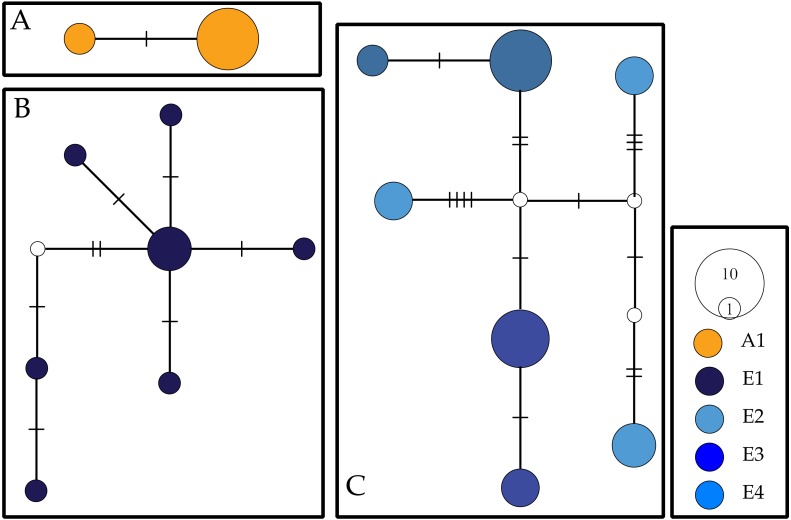
COI haplotype networks for Seychellois *Ligia*. Colors and locality IDs correspond with those use in all other Figures. Empty circles and hash marks represent unsampled (i.e., missing) haplotypes, while the size of circles is proportional to the frequency at which each haplotype was recovered. Each panel corresponds to networks separated by >5% differences.

For COI, we observed a total of 16 haplotypes divided into three networks (Fig. 2). Two haplotypes separated by two mutational steps were found solely in individuals collected at Aldabra. A second haplotype network contained all COI individuals collected from Cousine island (E1) and consisted of seven haplotypes separated by 2–9 steps. A third and final network consisted of the seven COI haplotypes recovered from Silhouette (E2) and Mahé (E3, E4) which were separated by 2–15 steps. We recovered two alleles for the NaK gene, with all *Ligia* individuals from the inner islands (E2–E4) sharing a single allele that diverged by 27 steps from an allele found in all the individuals from Aldabra (E1).

### Phylogenetic reconstructions

Our concatenated mitochondrial dataset included a total of 16 *Ligia* individuals: five from localities within the Seychelles, nine from other Indo-Pacific localities, and the two outgroups. The final concatenated alignment included 2025 nucleotides, of which 292 positions could not be confidently aligned and were excluded for the phylogenetic analyses (16S rDNA: 138; 12S rDNA: 154). Of the resulting 1733 nucleotides positions, 579 were parsimony informative. jModeltest identified a model consisting of three substitution rates (rate matrix: 012010; see jModeltest manual) as well as +F, +I, and +Γ parameters for our final concatenated mitochondrial dataset under the BIC, AIC, and AICc respectively. We applied this model in GARLI analyses; however, the more complex GTR +Γ model was applied in all other searches as the chosen model is not available in the other software packages (e.g., RAxML, Phycas). The use of GTR was justified as it was included in the 99% cumulative weight interval under all three selection criteria.

**Figure 3:**
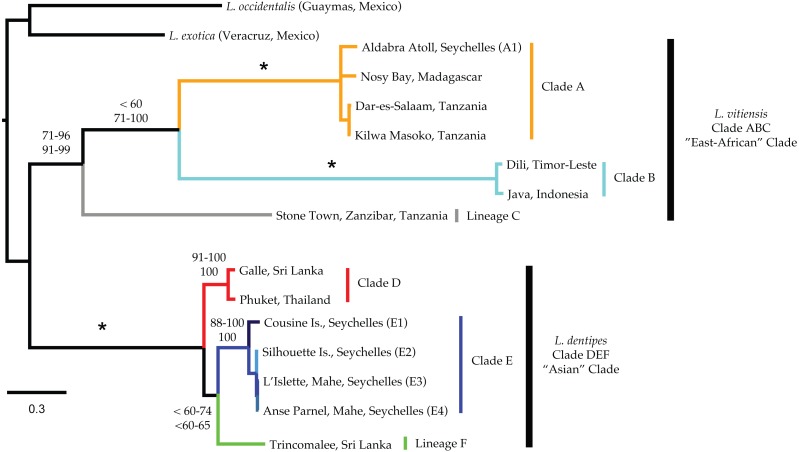
Majority rule consensus tree produced by Bayesian Analysis (GTR +Γ, unparitioned, Phycas) of the concatenated mitochondrial dataset of *Ligia* samples and outgroups included in this study. Numbers by nodes indicate the corresponding range of percent Bootstrap Support (BS; top) for Maximum likelihood; and Posterior Probabilities (PP; bottom) for Bayesian inference methods. Nodes receiving 100% for all methods are denoted with an * while an NS indicates less than 50% node support. Colors represent major lineages as discussed in text.

Mitochondrial phylogenetic reconstructions (Fig. 3) recovered a well-supported split (100 Bootstrap Support (BS) and Posterior Probability (PP)) between the *Ligia* specimens from the Indo-Pacific and the two outgroup taxa. Within the Indo-Pacific *Ligia*, we observed a split between two highly divergent lineages: an ‘East African’ clade (*Clade ABC*; BS: 71–96; PP: 91–99) and an ‘Asian’ clade (*Clade DEF*; BS: 100; PP: 100). COI K2P divergences between these two clades ranged from 21.8 to 27.8% with a mean divergence of 25.2%.

The ‘East African’ clade (*Clade ABC*) contained all specimens identified as *L. vitiensis* and included the localities of Aldabra Atoll in the Seychelles, Nosy Bay in Madagascar, three localities in Tanzania (i.e., Dar-Es-Salaam, Kilwa Masoko, and Stone Town), and two localities from the Indonesian Archipelago (Komodo, Indonesia and Dili, Timor-Leste). These localities formed three distinct lineages: (a) *Ligia* from Madagascar, mainland Tanzania, and Aldabra (Seychelles) were placed in the well-supported *Clade A* (light orange in all figures; BS: 100; PP: 100); (b) Indonesian archipelago *Ligia* were placed in *Clade B* (cyan in all figures; BS: 100; PP: 100); and (c) *Clade C* was composed of *Ligia* from Stone Town, Zanzibar, Tanzania (light grey in all figures). Although some analyses suggested a sister relationship between clades *A* and *B*, supports for this relationship was low in ML analyses (BS: <60) and highly variable in BI (PP: 71–100). COI K2P divergences between the three main lineages in the East African clade ranged from 7.12 to 9.09% (Table 3).

**Table 3:** Estimates of evolutionary divergence, as measured by Kimura 2-parameter distances, for main *Ligia* lineages from the study area and outgroups. When applicable minimum and maximum (top values) as well as average divergences (values in parentheses) are provided.

	*Clade A*	*Clade B*	*Clade C*	*Clade D*	*Clade E*	*Clade F*	*L. exotica*	*L. occidentalis*
*Clade A*	7.1–9.1% (4.3%)							
*Clade B*	22.9–24.7% (24.0%)	2.50%						
*Clade C*	25.8–28.1% (26.8%)	25.9–26.6% (26.2%)	N/A					
*Clade D*	25.3–27.1% (26.3%)	22.0–23.8% (22.8%)	25.9–27.2% (26.6%)	4.70%				
*Clade E*	24.3–27.6% (26.3%)	21.9–23.7% (22.1%)	24.7–25.4% (25.2%)	12.4–14.8% (13.7%)	0.5–4.7% (2.9%)			
*Clade F*	26.3–27.8% (27.3%)	23.3–23.75% (23.5%)	27.7%	13.3–14.2% (13.8%)	12.1–12.7% (12.3%)	N/A		
*L. exotica*	22.6–25.4% (23.4%)	25.7–25.7% (25.7%)	25.7%	25.9–28.1% (27.0%)	24.9–26.9% (25.9%)	26.3%	N/A	
*L. occidentalis*	27.5–28.3% (28.0%)	23.8–24.9% (24.3%)	24.6%	24.9%	19.0–20.9% (19.8%)	20.8%	25.6%	N/A

The ‘Asian’ clade (*DEF*) included all individuals identified as *L. dentipes* which were separated into three main lineages: (a) *Clade D* (red in all figures; BS: 91–100, PP:100) included *Ligia* from Galle (Sri Lanka) and Phuket (Thailand); (b) *Clade E* (blues in all figures; BS: 88–100; PP: 100) consisting of *Ligia* from the inner islands of Seychelles; and (c) *Clade F* (green in all figures) represented solely from *Ligia* collected in Trincomalee, Sri Lanka. As in *Clade ABC*, relationships within the ‘Asian’ clade were not well resolved. We observed a sister relationship between clades *E* and *F*; however, support values for this relationship were often low (BS: <60–74; PP: <60–65). COI K2P divergences between the three main lineages in the Asian clade ranged from 12.07 to 14.83% (Table 3). Divergences within the inner Seychelles locations (i.e., *Clade E*) ranged from 0.5 to 5.2% with all comparisons to the island of Cousine being above 4.5% (Table 4).

**Table 4:** Estimates of evolutionary divergence, as measured by Kimura 2-parameter distances, for *Ligia* localities from Seychelles inner island localities.

	E1	E2	E3	E4
E1	–			
E2	4.9%	–		
E3	5.1%	0.8%	–	
E4	5.2%	0.9%	0.5%	–

## Discussion

To date, two species of *Ligia* have been reported to inhabit intertidal habitats in the Seychelles archipelago: *L. dentipes* (Taiti, 2014) and *L. exotica* (Ferrara & Taiti, 1985). The former is considered to be endemic to coastlines in the north-eastern Indian Ocean, with confirmed records from the Nicobar and Andaman islands, the Maldives, Sri Lanka, and the Seychelles (Taiti, 2014 and references therein). *Ligia exotica*, on the other hand, is a species of possible East-Asian origin (LA Hurtado, 2010–2016, unpublished data) thought to have a cosmopolitan distribution due to human-aided introductions. These records, however, are based on morphological identifications, leaving in doubt their validity and whether they represent highly divergent genetic lineages as reported for *Ligia* species in other regions (Eberl et al., 2013; Hurtado, Mateos & Santamaria, 2010; Santamaria, Mateos & Hurtado, 2014; Santamaria et al., 2013; Taiti et al., 2003). By combining morphological identifications based on male sexual characteristics with molecular approaches to characterize *Ligia* individuals from both inner and outer islands in the Seychelles, we have determined that *Ligia* diversity in the Seychelles archipelago is underreported. Our phylogenetic reconstructions and morphological identifications suggest the presence in the Seychelles of highly divergent lineages belonging to at least two species complexes: *L. vitiensis* and *L. dentipes*. *Ligia* from the outer island of Aldabra represent a highly divergent lineage within a clade composed of *L. vitiensis* individuals from around the Indo-Pacific, while those from the inner islands of Cousin, Mahé, and Silhouette represent two highly divergent lineages within a clade composed of *L. dentipes* individuals from Asia. These findings are supported by male gonopod morphology and suggest that *Ligia* have colonized the Seychelles archipelago on at least two separate occasions.

Phylogenetic reconstructions place *Ligia* individuals collected at Aldabra Atoll, in *Clade ABC* with *L. vitiensis* individuals collected from East Africa and Madagascar. Despite the poorly resolved relationships within this clade, the geological history of Aldabra, oceanographic patterns in the region, and previous phylogeographic findings suggest a Malagasy or East African origin to be the most likely source of the *Ligia* populations in Aldabra. This outer island is a low lying atoll that originated some 20 Mya from coral reefs growing on volcanic seamounts (Plummer, 1995). The atoll does not appear to have experienced any connection to continental landmasses or other nearby-islands, but has undergone several periods of submersion and emergence, including what is thought to have been a complete submersion some 125,000 years ago (Braithwaite, Taylor & Kennedy, 1973). Such geological history would seem to preclude Aldabra as the place of origin for *Ligia* in the *ABC clade*. Colonizers instead appear more likely to have originated in either Madagascar or East Africa given the proximity of Aldabra to these continental landmasses (∼420 km from the coastline of Madagascar and ∼650 km from the East African coastline). Over-water dispersal events over long distances, although seemingly at odds with the biology of *Ligia*, have certainly occurred within the genus as indicated by the presence of endemic *Ligia* species in remote oceanic archipelagos such as Hawai‘i (Santamaria et al., 2013; Taiti et al., 2003) and the Marquesas Islands (Jackson, 1933). In the Seychelles, poorly-dispersing endemic taxa such as the Seychelles Tiger Chameleon *Archaius tigris* (Townsend et al., 2011) and the freshwater crab *Seychellum alluaudi* (Daniels, 2011) are thought to have originated by over-water dispersal.

*Ligia* individuals collected from the inner Seychelles islands of Cousin, Mahé, and Silhouette were placed in *Clade DEF* with *L. dentipes* individuals from Asia indicating either an Asian origin for these populations, or a Seychellois origin for *L. dentipes*. Although the relationships within *Clade DEF* are not well resolved, our findings suggest the Seychelles lineage to have evolved from an Asian ancestor: phylogenetic reconstructions place *Ligia* from the inner islands in *Clade E*, which is suggested to have split from an Asian ancestor (though poorly supported). Furthermore, the COI K2P divergences observed between *Ligia* from the inner islands and other *Ligia* from the region range from 12.07 to 27.62% (*L. dentipes*: 12.07–14.83% COI K2P; *L. vitiensis*: 21.85–27.62% COI K2P). These divergences would require mutation rates that are about six times lower than the mutation rate for COI reported for other marine isopods (2.5%/My; Ketmaier, Argano & Caccone, 2003) in order to match the age of the inner Seychelles islands (∼65 My; Plummer & Belle, 1995). These findings, though not conclusive, would suggest an Asian origin for *Ligia* from the inner islands.

The finding that *Ligia* from the inner islands are most closely related to those from Asia represent a rare, yet not exceptional, occurrence for Seychellois flora and fauna. Overall, fauna endemic to the Seychelles appears to largely be of an East African or Malagasy origin (Agnarsson & Kuntner, 2012 and references therein). Such findings are consistent with the closer proximity of these islands to East African and Madagascar than to Asian coastlines, and the predominant oceanic currents in the area. Nonetheless, an Asian affinity has been reported for several Seychellois organisms such as the Sooglossidae and Nasikabatrachidae anuran families (Biju & Bossuyt, 2003), coleopterans (Gerlach, 2009), and *Nepenthes* pitcher plants (Meimberg et al., 2001). Such affinity may be explained by Gondwanan organisms rafting on the Indian continent until its collision with Asia (Karanth, 2006) or by the over-water dispersal from Asia to the Seychelles or vice-versa. This latter mechanism has been suggested as the origin for a few terrestrial organisms from the Seychelles (Agnarsson & Kuntner, 2012 and references therein) and may explain the origin of *Ligia* in the inner islands. Our phylogeographic findings and morphological identifications coupled with the occurrence of *L. dentipes* across Indian Ocean archipelagos such as the Maldives (Taiti, 2014) indicate the possibility of a colonization of the Seychelles archipelago after rafting or “island-hopping” throughout the Indian Ocean; however, the poor support for relationships within *Clade DEF* indicates additional research remains needed to determine the origin of *Ligia* from the inner islands of the Seychelles.

Additional work is also required to determine whether *Ligia* from the Seychelles represent yet to be described cryptic species. We report levels of genetic divergence between sampled individuals from the Seychelles and other members of their clades that exceed those proposed for species-level divergences (COI K2P > 3%; Hebert et al., 2003) and largely match or exceed those reported from comparisons between valid *Ligia* species (Hurtado, Mateos & Santamaria, 2010; Santamaria, Mateos & Hurtado, 2014; Santamaria et al., 2013; Taiti et al., 2003). *Ligia* from Aldabra exhibit COI K2P divergences of ∼27% when compared to other *L. vitiensis* individuals from *Clade ABC*, while individuals from the inner islands show COI K2P divergences of ∼15% from *L. dentipes* individuals in *Clade DEF*. Lastly, individuals from Cousine Island exhibit COI K2P divergences of 4.5–5.3% when compared to other localities (Mahé and Silhouette) within the inner islands. Interestingly, this pattern is similar to those previously reported for Seychellois herpetofauna from the inner islands (Rocha, Harris & Posada, 2011; Rocha, Posada & Harris, 2013; Silva et al., 2010) and to reports of cryptic diversity for other *Ligia* species, including *L. occidentalis* (Eberl et al., 2013; Hurtado, Mateos & Santamaria, 2010), *L. hawaiensis* (Santamaria et al., 2013; Taiti et al., 2003), *L. baudiniana* (Santamaria, Mateos & Hurtado, 2014), *L. exotica* and *L. cinerascens* (Yin et al., 2013), and *L. oceanica* (Raupach et al., 2014). The sum of our findings thus suggest the presence of 2–3 putative cryptic species of *Ligia* in the Seychelles: Aldabra *Ligia* being one, and those of Cousine and Silhouette + Mahé representing one or two putative species.

Morphological inspections are needed to determine whether diagnostic characters exist that differentiate between these highly divergent lineages. Uncovering such differences may prove difficult, as previous morphological comparisons of highly divergent *Ligia* lineages within the same species complex suggest lineages to be nearly morphologically identical (Santamaria et al., 2016; Santamaria et al., 2013; Taiti et al., 2003). In such case, differences in other traits that have been proven useful to distinguish between cryptic species could be explored, including timing of reproduction (e.g., Villanueva, 2016), reproductive behaviors (e.g., Meguro et al., 2016), or molecular characters (e.g., Johnson et al., 2015). Our findings also suggest that additional lineages may occur in other *Ligia* populations across the Seychelles, particularly in yet to be sampled outer islands.

##  Supplemental Information

10.7717/peerj.3894/supp-1 Supplemental Information 1 Mitochondrial alignment for phylogenetic analyses

10.7717/peerj.3894/supp-2 Supplemental Information 2 Alignment for the NaK gene for Seychellois *Ligia*

10.7717/peerj.3894/supp-3 Supplemental Information 3 Alignment for COI gene for Seychellois *Ligia*

## Additional Information and Declarations

### Field Study Permissions

The following information was supplied relating to field study approvals (i.e., approving body and any reference numbers): Field collections in Seychelles were carried out under a collection permit approved by the Seychelles Bureau of Standards (Permit #A0157).

### DNA Deposition

The following information was supplied regarding the deposition of DNA sequences: The sequences have been uploaded to GenBank and accession numbers provided in Table 1. Alignments are provided as Supplemental Files.

### Data Availability

The following information was supplied regarding data availability: Newly produced sequences have been deposited in GenBank.
